# Beyond the auditory: anxiety bridges sleep disturbances and depressive symptoms to tinnitus handicap

**DOI:** 10.3389/fpsyt.2026.1830941

**Published:** 2026-05-22

**Authors:** Lijuan Fan, LiTing Fan, QiQi Wu, Jie Zhou, Liya Wu, Hantong Hu, Hong Gao

**Affiliations:** 1 Department of Comprehensive Rehabilitation, Zhejiang Rehabilitation Medical Center The Rehabilitation Hospital Area , The Third Affiliated Hospital of Zhejiang Chinese Medical University, Hangzhou, China; 2 School of Humanities and Law, Jiaxing University, Jiaxing, China; 3 Department of Acupuncture, Moxibustion and Massage, Wenzhou Central Hospital, Wenzhou, China; 4 Department of Acupuncture and Moxibustion, The Third Affiliated Hospital of Zhejiang Chinese Medical University, Hangzhou, China; 5 Department of Traditional Chinese Medicine, Pujiang Maternal and Child Health Center, Jinhua, China

**Keywords:** anxiety, depression, mediation analysis, sleep disorders, tinnitus

## Abstract

**Background:**

Tinnitus is closely associated with psychological factors including anxiety, depression, and sleep disturbances. However, how these variables interact to influence tinnitus severity remains poorly understood, warranting further exploration.

**Methods:**

This retrospective study analyzed data from 285 patients with tinnitus, utilizing the Tinnitus Handicap Inventory (THI), Self-Rating Depression Scale (SDS), Self-Rating Scale of Sleep (SRSS), and Self-Rating Anxiety Scale (SAS) as assessment instruments. Univariate and multivariate regression analyses were performed to identify psychosocial factors associated with THI scores. Structural equation modeling (SEM) was employed to examine the mediating role of anxiety—specifically, whether sleep disturbances and depression exacerbate tinnitus handicap indirectly through heightened anxiety. Subgroup analyses were further conducted to evaluate the stability of this psychological mediation pathway across different demographic and clinical subgroups (e.g., age, gender, disease duration).

**Results:**

Tinnitus severity was significantly positively correlated with scores on the SRSS, SDS, and SAS (all P < 0.05). Multivariate regression analysis identified age, sleep quality (SRSS), and anxiety (SAS) as independent predictors of increased Tinnitus Handicap Inventory (THI) scores. Structural equation modeling further confirmed that SAS partially mediated the relationship between SRSS and THI (mediation effect: 31.7%) and fully mediated the relationship between SDS and THI. Subgroup analyses revealed that the mediating effect of anxiety was more pronounced in middle-aged and elderly individuals, females, and patients with left-sided tinnitus.

**Conclusion:**

Anxiety serves as a central mediating mechanism linking sleep disturbances and depressive symptoms to tinnitus severity. The present findings demonstrate that anxiety not only directly exacerbates tinnitus-related handicap but also mediates the influence of sleep and mood disturbances on tinnitus distress. These findings underscore the critical role of anxiety in personalized tinnitus treatment.

## 1 Introduction

Tinnitus is increasingly recognized not merely as an auditory phenomenon, but as a complex psychosomatic condition in which emotional distress plays a pivotal role in determining disease burden. Clinically, tinnitus frequently co-occurs with anxiety, depression, and sleep disturbances (1–4). Although it is now widely recognized that tinnitus severity correlates with the aforementioned psychological factors (5–7), current understanding remains largely limited to these correlational findings. The intricate interactions among these psychological variables—and particularly the potential pathway-based relationships underlying them—remain poorly elucidated.

The neural underpinnings of these psychological interactions have been partially elucidated by neuroimaging evidence, which suggests that the persistence and perceptual salience of tinnitus may arise from aberrant interactions between auditory and non-auditory brain networks. At the level of the non-auditory network, Earlier studies proposed the abnormal interactions of three functional brain networks (8, 9): 1. The memory-emotion network (including the parahippocampal gyrus, amygdala, and other components of the limbic system, as well as the insula); 2. The attention-consciousness network (including the anterior/posterior cingulate, prefrontal cortex regions, subcallosal area, and precuneus); 3. The sensory-motor pathways (including the somatosensory ganglia, brainstem pathways, somatosensory cortex, and cerebellum). The differential engagement of these networks may underlie the clinical heterogeneity of tinnitus (10), particularly regarding its psychological comorbidities. For instance, anxiety-dominant tinnitus may primarily involve hyperactivation within the memory-emotion network (8).

Given the limited understanding of how psychological variables interact in tinnitus, the present study adopted an exploratory approach to examine the pathway-based relationships among anxiety, depression, sleep disturbances, and tinnitus severity using structural equation modeling.

## 2 Methods

### 2.1 Study population and data collection

A retrospective analysis was conducted on data collected through the Wenjuanxing electronic survey platform. This study included outpatients with tinnitus who visited the Tinnitus and Hearing Loss Clinic at the Third Affiliated Hospital of Zhejiang Chinese Medical University between March 2023 and March 2025.

### 2.2 Participants and ethics

Participants were (1) aged ≥18 years and enrolled irrespective of gender (2) clinically diagnosed with primary subjective tinnitus without any underlying organic pathology, regardless of disease duration and laterality, (3) tympanogram excludes type B or type C, and (4) capable of independently completing self-report questionnaires. Studies involving humans were approved by the Ethics Committee of the Third Affiliated Hospital of Zhejiang Traditional Chinese Medicine University (No.ZSLL-ZN-2022-046-01) and performed in compliance with relevant laws and institutional guidelines. Since the retrospective study analyzed only anonymized data, individual participants could not be identified, informed consent were therefore waived by the institutional review board.

### 2.3 Questionnaire design

The questionnaire was structured into three distinct sections. The demographic section (Section I) collected participants’ gender, age, marital status, and clinical history. Subsequent sections focused on tinnitus characteristics: Section II documented clinical parameters including symptom duration, laterality, etiological factors, and relevant medical comorbidities. Section III integrated audiometric data (pure-tone thresholds), psychoacoustic tinnitus profiling (pitch and loudness matching), and validated psychometric assessments incorporating standardized instruments: Tinnitus Handicap Inventory (THI), Self-Rating Sleep Scale (SRSS), Self-Rating Depression Scale (SDS), and Self-Rating Anxiety Scale (SAS).

### 2.4 Tinnitus handicap inventory

THI (11) quantifies tinnitus severity through three domains: Functional (11 items assessing physical/mental/social impacts), Emotional (9 items evaluating irritability/anxiety/depression), and Catastrophic (5 items measuring control/tolerance). Responses are scored as “Yes” (4), “Sometimes” (2), or “No” (0), with total scores (0-100) categorized into five severity levels: ≤16 (Grade I - slight), 18-36 (Grade II - mild), 38-56 (Grade III - moderate), 58-76 (Grade IV - severe), and ≥78 (Grade V - catastrophic) (12).

### 2.5 Zung’s self-rating anxiety scale

The SAS developed by Zung in 1971 (13), quantifies anxiety severity over the last week through 20 items. Each item is scored on a 4-point scale based on symptom frequency. Raw scores are converted to standardized values by multiplying the sum by 1.25, with final scores rounded to whole numbers. Clinical interpretation follows these thresholds: 50-59 (mild), 60-69 (moderate), and ≥70 (severe) anxiety.

### 2.6 Zung’s self-rating depression scale

Developed by Zung in 1965 (14), the SDS evaluates depressive symptoms over the past week with 20 items. Each item is scored on a 4-point scale based on symptom frequency. The scores are summed, multiplied by 1.25, and rounded to obtain the standard score. According to Chinese norms, the standard score cut-off is 53, with severity classified as follows: 53-62 (mild), 63-72 (moderate), and ≥73(severe).

### 2.7 Self-rating scale of sleep

SRSS was developed by Professor Li and was standardized with Chinese norms established through a national collaborative research consortium (15). The SRSS is a 10-item self-assessment tool designed to evaluate an individual’s sleep quality over the past month. Each item is rated on a 1-5 scale, yielding a total score ranging from 10 to 50, with higher scores indicating poorer sleep quality. The scale demonstrates acceptable reliability and validity.

### 2.8 Pure tone audiometry and tinnitus psychoacoustic measurements

PTA and TPM are standard audiological assessments for evaluating hearing ability and characterizing tinnitus. PTA determines the type and severity of hearing loss by measuring the minimum audible threshold intensity across frequencies (125–20, 000 Hz). TPM quantifies subjective tinnitus perception through three parameters: frequency matching, loudness matching, and minimum masking level. However, as this study focused on analyzing tinnitus characteristics (e.g., pitch and severity) and loudness matching has not demonstrated sufficient reliability and validity in assessing tinnitus (10), only frequency matching data were collected. The audiometer used in this study was the AC40 (Interacoustics, Denmark).

### 2.9 Sample size calculation

Sample size was calculated based on the requirement of at least 10 participants per variable in multivariate regression analysis, resulting in a minimum required sample of 200 participants.

### 2.10 Statistical analysis

Statistical analyses were conducted using IBM SPSS Statistics 25.0(IBM Corp., Armonk, NY). All tests were two-tailed, with statistical significance set at *p* ≤ 0.05. Structural equation modeling (SEM) path analysis was performed using AMOS 26.0 (IBM Corp., Armonk, NY).

Descriptive analyses were conducted for all variables. Normally distributed continuous data were expressed as mean ± standard deviation (SD), non-normally distributed data as median and interquartile range (IQR), and categorical/ordinal data as frequencies and percentages.

Based on the assumptions of normality and homogeneity of variance, continuous variables were analyzed using independent samples t-tests or ANOVA. Categorical variables were assessed using the chi-square test or Fisher’s exact test, as appropriate. Correlations between continuous variables were evaluated using Pearson’s correlation coefficient for normally distributed data and Spearman’s rank correlation for non-normally distributed data.

Univariate analyses were followed by multivariate linear regression models, with THI scores as dependent variables and tinnitus severity–related factors as independent variables. To avoid model overfitting, covariates in multivariate models were restricted to ensure at least 10 events per variable (16).

SEM was employed to explore mediation effects and inter-variable pathways, quantifying both direct and indirect effects. Subgroup analyses (e.g., by age, sex, and disease duration) were also performed, utilizing separate multivariate regression models adjusted for potential confounders. A minimum sample size of 50 was required for each subgroup analysis.

## 3 Result

### 3.1 Participants sample

**Figure 1:**
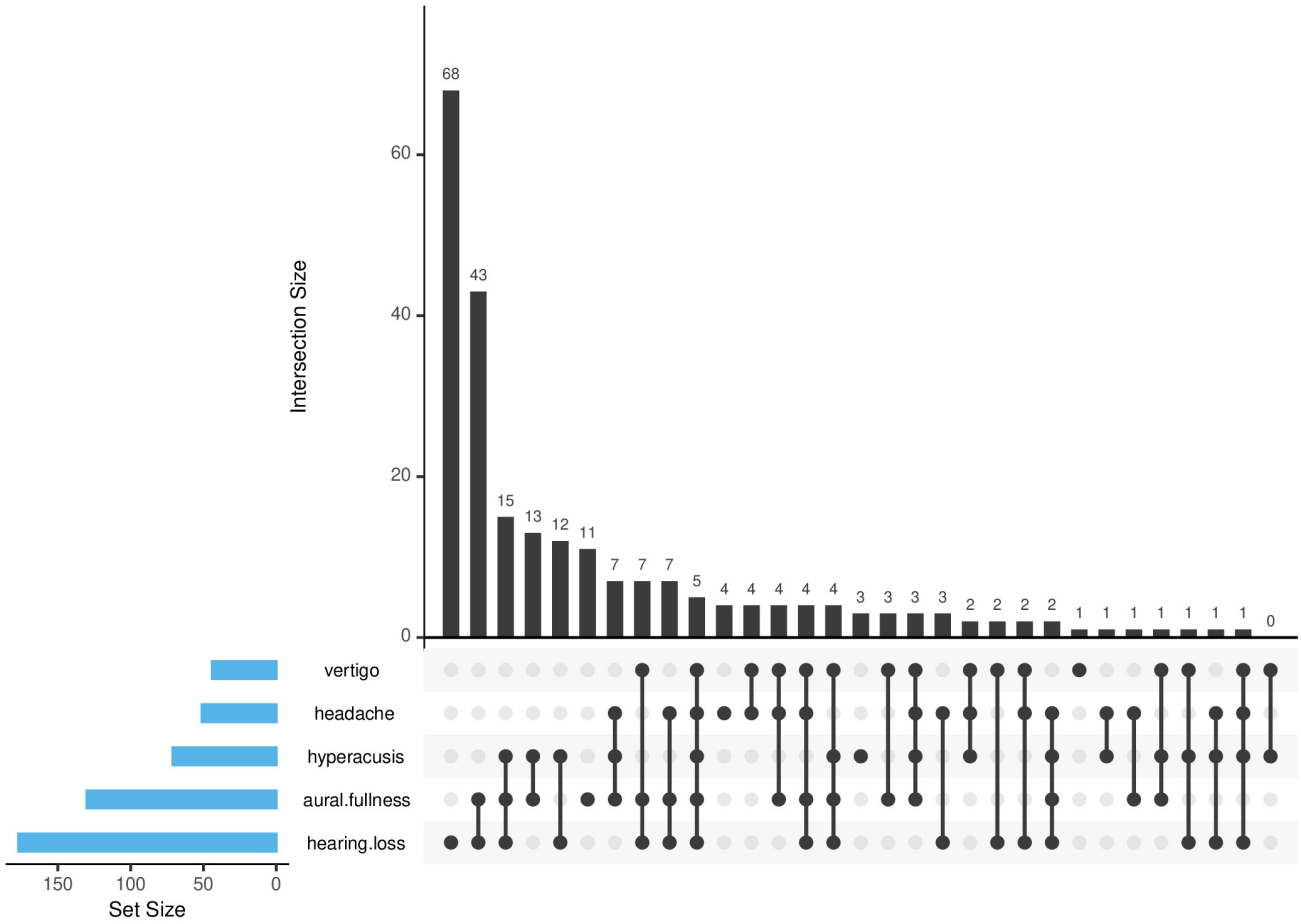
UpSet plot analysis of tinnitus-associated symptom clusters. A horizontal bar plot portraying the total elements in each set, a vertical bar plot indicating elements in corresponding intersections, and a matrix with connected dots delineating all intersection types among sets.

**Figure 2:**
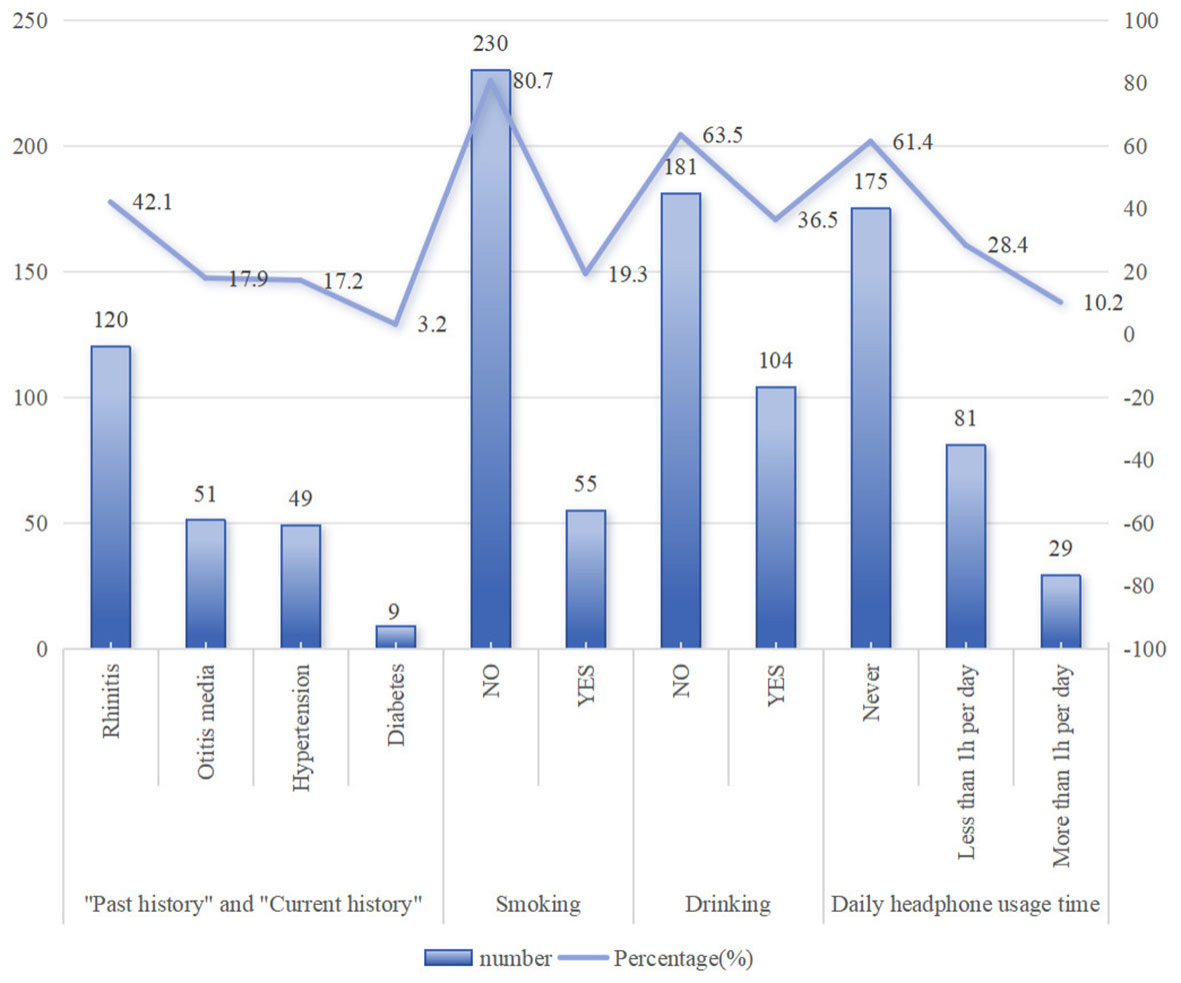
Frequency distribution plot of past medical history & personal and social history.

From March 2023 to March 2025, 285 tinnitus patients from the Tinnitus and Hearing Loss Clinic at the Third Affiliated Hospital of Zhejiang Chinese Medical University anonymously completed the initial assessment questionnaire. **Table 1** and **Figures 1**, **2** present the participants’ basic characteristics, past medical history & personal and social history, and tinnitus-associated symptoms.

**Table 1:** Characteristics of participants.

Variable	n(%)/Mean ± SD
Age Group	285(45.90 ± 12.48)
18y-44y	133(46.7%)
45y-59y	103(36.1%)
60y-	49(17.2%)
Gender	
Male	120(42.1%)
female	165(57.9%)
Duration^a^	
Acute	109(38.2%)
Subacute	25(8.8%)
Chronic	151(53.0%)
Location	
Left ear	82(28.8%)
Right ear	59(20.7%)
Bilateral	142(49.8%)
Cephalic	2(0.7%)
THI	285(45.36 ± 25.99)
Grade I	46(16.1%)
Grade II	69(24.2%)
Grade III	82(28.8%)
Grade IV	48(16.8%)
Grade V;	40(14.0%)
SRSS	285(24.69 ± 7.25)
SAS	285(47.02 ± 9.11)
Non-anxious	198(69.5%)
Mild	67(23.6%)
Moderate	13(4.5%)
Severe	7(2.4%)
SDS	285(51.66 ± 13.58)
Non-anxious	114(40.0%)
Mild	58(20.4%)
Moderate	99(34.7%)
Severe	14(4.9%)

a Acute, less than 3 months; Subacute, between 3months and 6months; Chronic, more than 6 months (33).

N, Number of Participants; SD, standard deviation; THI, Tinnitus Handicap Inventory; SRSS, Self-Rating Scale of Sleep; SAS, Zung’s Self-Rating Anxiety Scale; SDS, Zung’s Self-Rating Depression Scale.

### 3.2 Univariate correlation analysis of tinnitus

**Table 2** showed that there was a positive correlation between two of the four tables THI, SRSS, SDS, and SAS.

**Table 2:** Correlation coefficient matrix.

Variable	THI	SDS	SRSS
SDS	0.386***		
SRSS	0.377***	0.320***	
SAS	0.518***	0.551***	0.382***

***p < 0.001. THI, Tinnitus Handicap Inventory; SAS, Self-Rating Anxiety Scale; SDS, Self-Rating Depression Scale; SRSS, Self-Rating Scale of Sleep.

### 3.3 Risk factor analysis for tinnitus severity

A multiple linear regression model was constructed using forward selection method, with THI as the dependent variable and incorporated the following variables: duration, SRSS, drinking, daily headphone usage time, headache, aural fullness, gender, SDS, hyperacusis, age, and SAS (**Table 3**). Four significant predictors were found. Age demonstrated a statistically significant positive association with THI scores (β=0.25, t=2.25, *p* < 0.05), indicating a 0.25-point increase in THI per additional year of age. Drinking status exhibited a significant negative association (β=-5.995, t=-2.13, *p* < 0.05), with drinkers showing 5.995-point lower THI scores compared to non-drinkers. Both SRSS and SAS scores were strongly positively correlated with THI: each 1-point increase in SRSS corresponded to a 0.7-point elevation in THI (β=0.7, t=3.69, *p* < 0.001), while each 1-point increase in SAS was associated with a near 1-point rise in THI (β=0.996, t=5.517, *p* < 0.001). Variables such as daily headphone usage time, headache, and aural fullness did not reach statistical significance in the final model. All models showed significant overall fit (*p* < 0.001) and no collinearity (VIF<1.5).

**Table 3:** Multivariate logistic regression analysis of determinants associated with tinnitus handicap inventory(THI).

Model	Unstandardized cofficients		Standardized cofficients	T	Sig.	Collinearity statistics	
	B	Std.Error	Beta			Tolerance	VIF
(Constant)	-27.262	10.415		-2.617	0.009		
Age	0.25	0.111	0.121	2.251	0.025*	0.796	1.256
Drinking	-5.995	2.819	-0.112	-2.127	0.034**	0.826	1.211
SRSS	0.7	0.19	0.196	3.688	0.000***	0.806	1.241
SAS	0.966	0.175	0.341	5.517	0.000***	0.599	1.669
Hyperacusis	8.518	3.06	0.145	2.783	0.006**	0.845	1.184
Adjusted R^2^	0.352						
F	15.004, *p* < 0.001						

Predictor Variable:(Constant), Duration, SRSS, Drinking, Daily headphone usage time, Headache, Aural fullness, Gender, SDS, Hyperacusis, Age, SAS.

*p < 0.05, **p < 0.01, ***p < 0.001. Std., standard; Sig., Significance Level;VIF, Variance Inflation Factor; SRSS, Self-Rating Scale of Sleep; SAS, Zung’s Self-Rating Anxiety Scale; SDS, Zung’s Self-Rating Depression Scale.

Age, SRSS, and SAS were identified as significant predictors of tinnitus severity, while drinking status was identified as a significant negative predictor (i.e., a protective factor), through THI-based linear regression models. Notably, drinking status in this study was recorded as a trichotomous variable (Yes/Previously drank but quit/No), reflecting whether participants reported regular alcohol consumption; the specific quantity of alcohol consumed was not assessed.

### 3.4 Structural equation model

Given the widely acknowledged association between depression and tinnitus, SEM was developed to elucidate the interrelationships among assessment scales.

**Figure 3:**
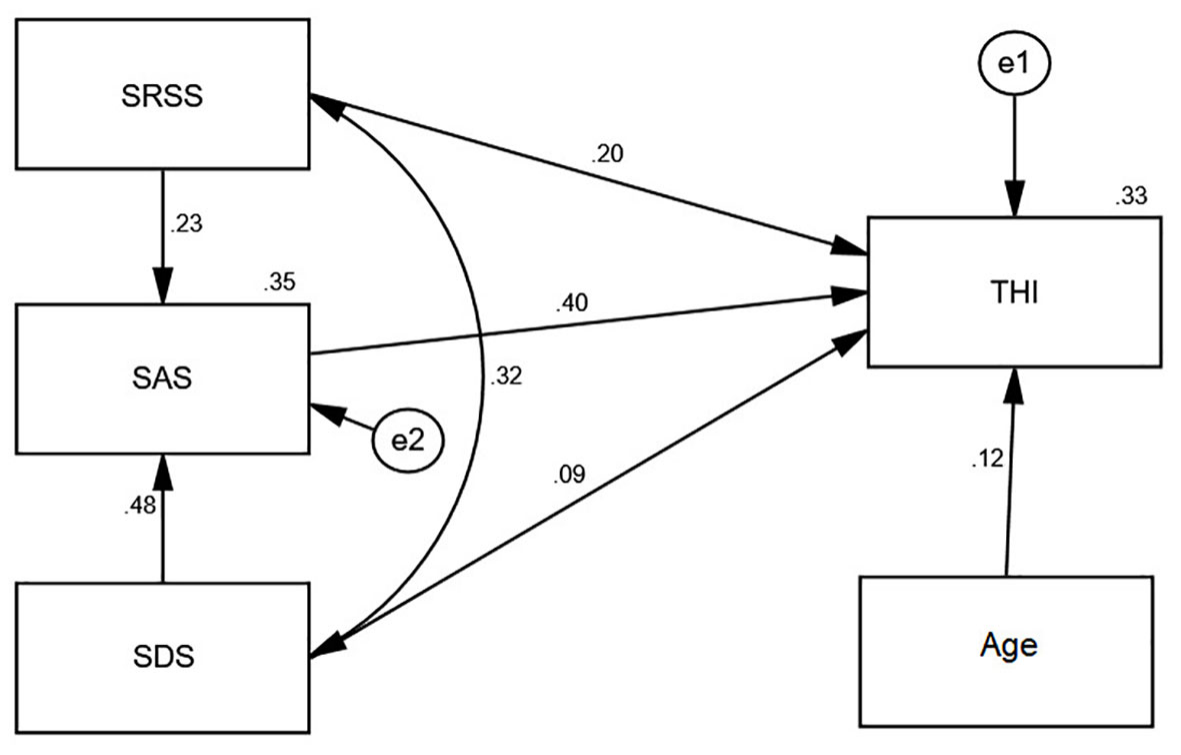
Structural equation model with standardized estimates. Rectangles represent observed variables, circles indicate residual errors. Solid arrows indicate significant paths (p<0.05). Standardized coefficients (β) shown. THI, Tinnitus Handicap Inventory; SRSS, Self-Rating Scale of Sleep; SAS, Zung’s Self-Rating Anxiety Scale; SDS, Zung’s Self-Rating Depression Scale.

SEM with standardized estimates are presented in **Figure 3**, and **Table 4** showed the goodness-of-fit indices. The model demonstrated excellent fit with the following metrics: χ²/df = 2.284 (below the threshold of 3), GFI = 0.991, AGFI = 0.953 (both exceeding the 0.8 benchmark), NFI = 0.975, TLI = 0.951, CFI = 0.985 (all surpassing the 0.9 cutoff), and RMSEA = 0.067 (within the acceptable range of ≤0.08). All indices satisfied established fit criteria, validating the model’s appropriateness for path analysis.

**Table 4:** Goodness-of-fit indices for the structural equation model.

Index	X²/df	GFI	AGFI	NFI	TLI	CFI	RMSEA
Value	2.284	0.991	0.953	0.975	0.951	0.985	0.067
Recommended Threshold	<3	>0.8	>0.8	>0.9	>0.9	>0.9	<0.08
Met Criteria?	Yes	Yes	Yes	Yes	Yes	Yes	Yes

X²/df, Chi-square divided by degrees of freedom; GFI, goodness of fit index; AGFI, adjusted goodness of fit index; NFI, Normed Fit Index; TLI, Tucker-Lewis Index; CFI, Comparative Fit Index; RMSEA, Root mean square error of approximation.

Standardized regression coefficients and variance parameter estimates of the SEM are detailed in **Table 5** Path Coefficient Estimates and Significance Tests in the Structural Equation Model: 1) The path coefficient from SRSS to THI was 0.196 (CR = 3.700, *p* < 0.001), demonstrating a statistically significant positive effect of SRSS on THI. 2) The path coefficient from SAS to THI was 0.398 (CR = 6.621, *p* < 0.001), demonstrating a highly significant positive effect of SAS on THI. 3) The path coefficient from SDS to THI was 0.095 (CR = 1.613, *p* > 0.05), indicating no statistically significant positive effect of SDS on THI. 4) The path coefficient from Age to THI was 0.121 (CR = 2.505, *p* < 0.05), illustrating a statistically significant positive effect of Age on THI. 5) The path coefficient from SRSS to SAS was 0.228 (CR = 4.520, *p* < 0.001), showing a highly significant positive effect of SRSS on SAS. 6) The path coefficient from SDS to SAS was 0.478 (CR = 9.461, *p* < 0.001), revealing a highly significant positive effect of SDS on SAS.

**Table 5:** Path coefficient estimates and significance tests in the structural equation model.

Path			Estimate	S.E.	C.R.	*P*
THI	←	SRSS	0.196	0.189	3.7	0.000***
THI	←	SAS	0.398	0.171	6.621	0.000***
THI	←	SDS	0.095	0.113	1.613	0.107
THI	←	Age	0.121	0.101	2.505	0.012*
SAS	←	SRSS	0.228	0.063	4.52	0.000***
SAS	←	SDS	0.478	0.034	9.461	0.000***

*P < 0.05, ***P < 0.001, S.E, Standard Error; C.R, Critical Ratio. THI, Tinnitus Handicap Inventory; SRSS, Self-Rating Scale of Sleep; SAS, Zung’s Self-Rating Anxiety Scale; SDS, Zung’s Self-Rating Depression Scale.

To investigate potential mediation effects within the significant pathways, we employed the bootstrap method in AMOS 26.0 with 5, 000 resamples and 95% bias-corrected confidence intervals (95% BCCI). The standardized specific indirect effects were computed by implementing pathway constraints through the software’s built-in syntax. As shown in **Table 6**: 1)The specific indirect effect of SRSS → SAS → THI was 0.091 (95% BCCI[0.050, 0.136]). The exclusion of zero from the confidence interval confirms the significant mediating role of SAS in the SRSS-THI relationship. 2)The indirect pathway SDS → SAS → THI demonstrated a mediation effect of 0.190 (95% BCCI[0.126, 0.255]). The absence of zero within the interval indicates statistically significant mediation by SAS in linking SDS to THI.

**Table 6:** Mediation analysis results with bootstrap confidence intervals (SAS).

Mediation pathway	Estimate	Lower	Upper	*P*
SRSS→SAS→THI(indirect effect)	0.091	0.050	0.136	0.000***
SDS→SAS→THI(indirect effect)	0.190	0.126	0.255	0.000***
SRSS→THI(direct effect)	0.196	0.087	0.301	0.000***
SRSS→THI(total effect)	0.287	0.177	0.393	0.000***
SDS→THI(direct effect)	0.095	-0.019	0.209	0.095
SDS→THI(total effect)	0.285	0.183	0.386	0.000***

***P < 0.001. THI, Tinnitus Handicap Inventory; SRSS, Self-Rating Scale of Sleep; SAS, Zung’s Self-Rating Anxiety Scale; SDS, Zung’s Self-Rating Depression Scale.

Age, SRSS, and SAS demonstrated significant positive correlations with THI scores. SDS exerted a positive influence on THI scores fully through the mediating effect of SAS, while SAS also mediated 31.7% of the total effect of SRSS on THI.

### 3.5 Subgroup analyses

Subgroup analyses stratified by gender, age, and disease duration (**Table 7**) revealed distinct patterns in risk and protective factors for tinnitus severity (THI scores). All models showed significant overall fit (*p* < 0.001) and no collinearity (VIF<1.5).

**Table 7:** Multivariate regression of factors associated with tinnitus severity (THI) stratified by gender, age, duration and ear.

Group		Model	Unstandardized cofficients		Standardized cofficients	T	Sig.	Collinearity statistics	
			B	Std. error	Beta			Tolerance	VIF
Gender	Male	(constant)	-29.761	9.756		-3.051	0.003		
		Hyperacusis	17.213	5.410	0.237	3.182	0.002**	0.939	1.065
		SRSS	1.548	0.311	0.403	4.976	0.000***	0.795	1.258
		SAS	0.799	0.216	0.298	3.693	0.000***	0.802	1.246
		Adjusted R^2^	0.446						
		F	15.233, *p* < 0.001						
	Female	(constant)	-33.433	10.660		-3.136	0.002		
		Age	0.398	0.126	0.207	3.149	0.002**	0.979	1.022
		Hyperacusis	8.338	3.458	0.163	2.411	0.017*	0.924	1.082
		Drinking	-8.186	4.120	-0.131	-1.987	0.049*	0.976	1.024
		SAS	1.263	0.183	0.467	6.891	0.000***	0.923	1.083
		Emotional agitation	9.507	4.160	0.152	2.285	0.024*	0.957	1.044
		Adjusted R^2^	0.355						
		F	17.716, *p* < 0.001						
Age	Young	(constant)	-26.555	8.472		-3.134	0.002		
		SAS	0.922	0.181	0.381	5.101	0.000***	0.807	1.240
		SRSS	0.937	0.280	0.261	3.346	0.001***	0.737	1.357
		Emotional agitation	15.375	5.390	0.206	2.852	0.005**	0.857	1.166
		Hyperacusis	9.996	4.016	0.174	2.489	0.014*	0.918	1.089
		Hypertension	10.813	4.668	0.157	2.316	0.022*	0.980	1.020
		Adjusted R^2^	0.452						
		F	21.133, *p* < 0.001						
	Middle-Aged and Elderly^a^	(constant)	-45.597	13.262		-3.438	0.001		
		SAS	1.449	0.226	0.456	6.425	0.000***	0.936	1.068
		Hyperacusis	10.476	4.210	.177	2.488	0.014*	0.926	1.079
		Daily headphone usage time	-7.402	2.810	-0.182	-2.635	0.009**	0.989	1.011
		Adjusted R^2^	0.303						
		F	21.447, *p* < 0.001						
Duration	Acute and subacutea	(constant)	-41.070	12.168		-3.375	0.001		
		SAS	1.011	0.213	0.360	4.752	0.000***	0.828	1.208
		Age	0.604	0.147	0.286	4.100	0.000***	0.972	1.029
		Hyperacusis	11.542	3.972	0.208	2.906	0.004**	0.927	1.079
		SRSS	0.563	0.274	0.155	2.051	0.042*	0.829	1.207
		Drinking	-7.454	3.750	-0.140	-1.988	0.049*	0.960	1.042
		Adjusted R^2^	0.370						
		F	16.599, *p* < 0.001						
	Chronic	(constant)	-32.424	9.034		-3.589	0.000		
		SAS	1.313	0.200	0.458	6.560	0.000***	0.834	1.200
		SRSS	0.654	0.243	0.188	2.693	0.008**	0.831	1.203
		Daily headphone usage time	-5.950	2.552	-0.150	-2.332	0.021*	0.981	1.020
		Emotional agitation	13.306	4.919	0.179	2.705	0.008**	0.923	1.083
		Adjusted R^2^	0.391						
		F	20.241, *p* < 0.001						
Location	Left	(constant)	-21.129	11.18		-1.89	0.063		
		SAS	1.565	0.231	0.576	6.764	0.000***	0.977	1.023
		Daily headphone usage time	-11.718	3.426	-0.293	-3.42	0.001***	0.966	1.035
		Drinking	-9.945	4.729	-0.177	-2.103	0.039*	0.995	1.005
		Adjusted R^2^	0.455						
		F	16.083, *p* < 0.001						
	Right	(constant)	-26.676	15.149		-1.761	0.084		
		SAS	0.94	0.303	0.333	3.107	0.003**	0.806	1.241
		SRSS	1.762	0.404	0.466	4.356	0.000***	0.809	1.236
		Duration	-5.953	2.846	-0.207	-2.091	0.041*	0.946	1.057
		Adjusted R^2^	0.491						
		F	17.682, *p* < 0.001						
	Bilateral	(constant)	-7.713	10.767		-0.716	0.475		
		SAS	1.143	0.219	0.396	5.219	0.000***	0.893	1.12
		Drinking	-8.017	3.756	-0.157	-2.134	0.035*	0.945	1.058
		Hyperacusis	11.355	4.199	0.201	2.705	0.008**	0.931	1.075
		Adjusted R^2^	0.282						
		F	18.334, *p* < 0.001						

a, The elderly group was merged with the middle-aged group for analysis because of limited sample size;.

b, The subacute group was merged with the acute group for analysis because of limited sample size.

*p < 0.05, **p < 0.01, ***p < 0.001. Std., standard; Sig., Significance Level;VIF, Variance Inflation Factor; SRSS, Self-Rating Scale of Sleep; SAS, Zung’s Self-Rating Anxiety Scale; SDS, Zung’s Self-Rating Depression Scale.

In gender-based analysis, hyperacusis and anxiety (SAS) were significantly and positively correlated with THI in both males and females, with hyperacusis exhibiting a stronger effect in males (B = 17.218, *p* < 0.05) and SAS showing a greater impact in females (B = 1.263, *p* < 0.001). Additionally, poor sleep quality (SRSS) was a male-specific risk factor (B = 1.548, *p* < 0.001), while age and emotional agitation increased THI across all groups, and alcohol consumption emerged as a protective factor (B=-8.186, *p* = 0.049).

In age-stratified analysis, anxiety (SAS) and hyperacusis significantly increased THI in both younger and older adults, but their effects were stronger in older adults (SAS: B = 1.449 vs. 0.922; hyperacusis: B = 10.476 vs. 9.996). Younger adults were more susceptible to sleep disturbances (SRSS: B = 0.937, *p* = 0.001), emotional agitation (B = 15.375, *p* = 0.005), and hypertension (B = 10.813, *p* = 0.022), whereas prolonged headphone use reduced THI in older adults (B=-7.402, *p* = 0.009).

Disease duration analysis further highlighted that anxiety (SAS) and poor sleep quality (SRSS) consistently worsened THI in both acute/subacute and chronic phases, with stronger effects in chronic patients (SAS: B = 1.313 vs. 1.011, *p* < 0.001; SRSS: B = 0.654 vs. 0.563, *p* < 0.05). Acute/subacute-phase patients were influenced by age (B = 0.604, *p* < 0.001) and hyperacusis (B = 11.542, *p* = 0.004), with alcohol as a protective factor (B=-7.454, *p* = 0.049). In contrast, chronic-phase patients faced higher risks from emotional agitation (B = 13.306, *p* = 0.008), while headphone use showed protective effects (B=-5.950, *p* = 0.021).

Multiple linear regression analyses were performed to identify risk factors in patients with tinnitus comorbid with hearing loss, anxiety(SAS), or depression(SDS) (**Table 8**). Age demonstrated significant associations across all groups, with the strongest correlation observed for SAS (B = 0.561, *p* = 0.001). THI scores in patients with hearing loss were significantly associated with SAS (B = 1.040, *p* < 0.001), hyperacusis (B = 10.391, *p* = 0.007), and SRSS (B = 0.687, *p* = 0.005), explaining 42.1% of variance (Adjusted R²). In SAS and SDS groups, SDS (B = 1.050, *p* < 0.001) and SAS (B = 1.384, *p* < 0.001) were the strongest predictors, respectively. Aural fullness was linked to THI in tinnitus patients with anxiety (B = 12.845, *p* = 0.01), while SRSS (B = 0.766, *p* = 0.002) and hyperacusis (B = 9.141, *p* = 0.016) showed associations with THI in those with comorbid depression. All models showed significant overall fit (*p* < 0.001) and no collinearity (VIF<1.5).

**Table 8:** Multivariate linear regression analysis of factors associated with tinnitus severity (THI) in patients with comorbid hearing loss, anxiety (SAS), or depression (SDS).

Group	Model	Unstandardized cofficients		Standardized cofficients	T	Sig.	Collinearity statistics	
		B	Std. error	Beta			Tolerance	VIF
Hearing loss	(constant)	-25.040	11.635		-2.152	0.033		
	SAS	1.040	0.188	0.374	5.527	0.000***	0.743	1.345
	Hyperacusis	10.391	3.814	0.165	2.724	0.007**	0.933	1.072
	SRSS	0.687	0.243	0.186	2.832	0.005**	0.793	1.260
	Age	0.338	0.143	0.150	2.357	0.020*	0.842	1.188
	Adjusted R^2^	0.421						
	F	20.57, *p* < 0.001						
Anxiety (SAS)	(constant)	-34.424	15.485		-2.223	0.029		
	SDS	1.050	0.223	0.432	4.714	0.000***	0.943	1.061
	Age	0.561	0.165	0.306	3.408	0.001***	0.983	1.017
	Aural fullness	12.845	4.839	0.243	2.655	0.010**	0.945	1.058
	Adjusted R^2^	0.319						
	F	14.457, *p* < 0.001						
Depression (SDS)	(constant)	-53.367	14.158		-3.769	0.000		
	SAS	1.384	0.232	0.429	5.973	0.000***	0.779	1.284
	SRSS	0.766	0.238	0.222	3.222	0.002**	0.850	1.177
	Age	0.506	0.145	0.239	3.498	0.001***	0.861	1.162
	Hyperacusis	9.141	3.741	0.162	2.443	0.016*	0.919	1.088
	Adjusted R^2^	0.365						
	F	16.140, *p* < 0.001						

*p < 0.05, **p < 0.01, ***p < 0.001. Std., standard; Sig., Significance Level;VIF, Variance Inflation Factor; SRSS, Self-Rating Scale of Sleep; SAS, Zung’s Self-Rating Anxiety Scale; SDS, Zung’s Self-Rating Depression Scale.

## 4 Discussion

This study examined the relationship between psychological factors and tinnitus severity in 285 patients using regression analysis and structural equation modeling. Results indicated that anxiety and sleep disturbance were independent predictors of tinnitus severity, with anxiety fully mediating the relationship between depression and tinnitus.

Notably, anxiety is identified as the core influencing factor. Depression (SDS) affects THI entirely through the mediation of anxiety (with an indirect effect accounting for 66.67%), indicating that anxiety might be the “bridge” mechanism linking depression and tinnitus. This suggests that interventions targeting anxiety reduction, such as cognitive behavioral therapy or mindfulness-based stress reduction, may attenuate tinnitus severity, even in patients with comorbid depression. Clinically, this might imply prioritizing anxiety management in patients presenting with both depressive symptoms and tinnitus. Of note, the co-occurrence of tinnitus and anxiety may stem from dysregulation within the memory-emotion network, where the limbic system serves as a functional hub (17). Central to this interplay is the amygdala, which serves as a critical mediator for emotional and other behavioral responses to sensory stimuli across all senses (18). Dynamic connections between the limbic system and the primary auditory regions highlight the importance of the auditory-limbic system interaction in tinnitus (19). Additionally, the hypothalamic-pituitary-adrenal (HPA) axis may also explain the comorbidity of tinnitus and anxiety disorders (17). Chronically elevated cortisol levels impair hippocampal neurogenesis while sensitizing the amygdala, thereby concurrently driving anxiety symptoms and enhancing tinnitus-related distress (5).

Moreover, sleep disorders (SRSS) influence tinnitus through both the mediation of anxiety (31.7%) and an independent physiological pathway, revealing the “sleep-anxiety-tinnitus” vicious cycle. The underlying mechanisms and contributing factors of insomnia in tinnitus patients are not yet fully understood. Some researchers proposed that tinnitus and sleep disturbances may stem from overlapping physiological processes, such as sympathetic hyperactivity-induced hyperarousal (20). Other researchers emphasize the role of maladaptive cognitive patterns and emotional distress in perpetuating both conditions (21). Similarly the precise mechanisms linking sleep disorders and anxiety, however, remain incompletely understood and could involve disruptions in emotional regulation pathways, cognitive dysfunction, and sleep deprivation-induced circadian dysregulation (22). For tinnitus patients with sleep disorders patients, our findings suggest that therapies targeted anxiety concurrently with sleep hygiene interventions may break the cycle and improve prognosis. Furthermore, screening for and addressing specific maladaptive cognitive patterns related to both sleep and tinnitus perception could be beneficial.

Subgroup analyses revealed that anxiety exhibited higher sensitivity among middle-aged and elderly individuals, females, and subjects with left-sided tinnitus. These disparities likely stem from biological aging-related molecular changes that impact anxiety-related psychological processes (23–26), gender-specific neuroendocrine profiles and sociocultural pressures (27) and heightened right hemisphere activation in response to left ear tinnitus (28).These subgroup differences suggest that the role of anxiety in tinnitus is population-specific, necessitating individualized adjustments to intervention strategies. Of note, drinking status emerged as a significant negative predictor of THI scores in the overall regression model and in several subgroup analyses, suggesting a possible protective or attenuating role of alcohol consumption on tinnitus-related handicap. This finding should be interpreted with caution and should not be regarded as evidence supporting alcohol consumption as a treatment for tinnitus. The present study recorded drinking status as a trichotomous variable (Yes/Previously drank but quit/No) reflecting self-reported regular alcohol consumption, without quantifying the type or amount of alcohol consumed; therefore, dose-dependent effects could not be assessed. It is possible that moderate alcohol consumption produces transient anxiolytic effects that temporarily reduce tinnitus distress (29–32), or alternatively, that this finding reflects unmeasured confounding factors. The potential for self-medication behavior — whereby individuals with more distressing tinnitus may reduce or avoid alcohol use — cannot be excluded in this cross-sectional design. Therefore, future longitudinal studies should collect detailed information on alcohol quantity and drinking patterns to better characterize this relationship.

### 4.1 Clinical implications

Taken together, our findings have several important clinical implications. First, the central role of anxiety as a mediator suggests that anxiety screening and management should be prioritized in tinnitus treatment protocols. Second, subgroup analyses indicate that tailored strategies based on factors such as age and gender may enhance therapeutic efficacy by targeting the predominant psychological pathways underlying tinnitus distress in different patient populations.

In terms of management, these findings support the integration of psychological interventions into routine tinnitus care. Specifically, cognitive behavioral therapy (CBT) has demonstrated efficacy in reducing anxiety and improving tinnitus-related quality of life, and may be particularly beneficial given the mediating role of anxiety identified in this study. For patients presenting with comorbid sleep disturbances, targeted interventions such as cognitive behavioral therapy for insomnia (CBT-I) may additionally help to disrupt the “sleep-anxiety-tinnitus” cycle. A multidisciplinary approach incorporating audiological management alongside psychological support is therefore recommended for comprehensive tinnitus rehabilitation.

### 4.2 Limitations

Several limitations should be acknowledged. First, the study is observational cross-sectional design, which precludes the establishment of causal relationships between variables, so longitudinal cohort studies are required to validate temporal associations. Second, the reliance on self-reported questionnaires risks recall bias and subjective interpretation errors, necessitating integration of objective biomarkers (e.g., neuroimaging or biochemical markers) to enhance reliability. Third, single-center sampling introduces selection bias, potentially excluding mild untreated cases and the elderly population who have difficulty with digital survey formats due to limited digital literacy.

Future studies should adopt multicenter stratified sampling and mixed-mode data collection (digital + paper-based) to improve generalizability. Longitudinal cohort studies are required to validate temporal associations and establish causality, while the integration of objective biomarkers (e.g., neuroimaging or biochemical markers) could enhance reliability.

## 5 Conclusion

The present study identifies anxiety as a core mediator linking sleep disturbances and depressive symptoms to tinnitus severity. While tinnitus is often conceptualized as an auditory phenomenon, our findings underscore that its associated disability is predominantly shaped by psychological factors rather than clinical characteristics alone. Specifically, anxiety not only directly contributes to greater tinnitus handicap but also serves as a critical mediator through which poor sleep and depression exert their effects on tinnitus-related distress. This mediation model highlights the importance of targeting anxiety in clinical interventions, suggesting that psychological treatments—particularly those addressing maladaptive emotional responses such as anxiety—may be more effective in reducing tinnitus burden than approaches focused solely on auditory features. Furthermore, subgroup variations in this pathway indicate that psychological profiles differ across patient populations, supporting need for personalized intervention measures.

## Data Availability

The raw data supporting the conclusions of this article will be made available by the authors, without undue reservation.
