# Supplementary material for: Beyond the auditory: anxiety bridges sleep disturbances and depressive symptoms to tinnitus handicap
Source: Front Psychiatry. 2026 May 22;17:1830941. doi: 10.3389/fpsyt.2026.1830941 (PMC13236688; doi:10.3389/fpsyt.2026.1830941)
Supplement: Supplementary file 1 [file DataSheet1.pdf]

耳鸣患者调查及自测表

第 1 题　您的治疗状态？　[单选题]

选项	
初诊	
已经完成一个疗程治疗	
已经完成两个疗程治疗	
已经完成两个疗程以上仍持续治疗	
治疗结束后 1 个月随访	
治疗结束后 3 个月随访	

第 2 题　您的年龄?　[填空题]

第 3 题　您的具体治疗次数？　[填空题]

第 4 题　您的性别?　[单选题]

选项	
男	
女	

第 5 题　您的婚姻状况?　[单选题]

选项	
未婚	
已婚	
离异	
丧偶	

第 6 题　您发病至今多久了?　[单选题]

选项	
小于 2 周	
2 周-1 个月	
1-3 个月	
3-6 个月	
7 个月~1 年	
1~5 年	
6-10 年	
10 年以上（>10 年）	

第 7 题　您是单耳耳鸣还是双耳耳鸣?　[单选题]

选项	
无耳鸣	
单耳耳鸣	
双耳耳鸣	

第 8 题　您目前是否有耳聋症状?　[单选题]

选项	
是	
否	

第 9 题　您目前是否有眩晕症状?　[单选题]

选项	
是	
否	

第 10 题　您是否有头痛症状?　[单选题]

选项	
是	
否	

第 11 题　您目前是否有耳闷耳胀症状？　[单选题]

选项	
是	
否	

第 12 题　您是否有听觉过敏?　[单选题]

选项	
是	
否	

第 13 题　您是否曾患或现患鼻炎？　[单选题]

选项	
是	
否	

第 14 题　您是否曾患或现患中耳炎？　[单选题]

选项	
是	
否	

第 15 题　您是否曾患或现患高血压?　[单选题]

选项	
是	
否	

第 16 题　您是否曾患或现患糖尿病？　[单选题]

选项	
是	
否	

第 17 题　您是否曾吸烟或现有吸烟习惯?　[单选题]

选项	
从未	
曾有，已戒	
现在仍有	

第 18 题　您是否曾饮酒或现有饮酒习惯?　[单选题]

选项	
从未	
曾有，已戒	
现在仍有	

第 19 题　您佩戴耳机的时长?　[单选题]

选项	
从不佩戴耳机	
平均每日佩戴耳机时长≤1 小时	

平均每日佩戴耳机时长超过 1 小时	

第 20 题　症状出现的诱因？　[多选题]

选项	
劳累	
上呼吸道感染	
情绪激动	
噪声刺激	
其他	
无明显诱因	

第 21 题　您是否长期处于噪声环境?　[单选题]

选项	
是	
否	

第 22 题　发病以来，您有无使用过药物治疗?　[多选题]

选项	
无	
有，曾用激素类药物（如地塞米松等），已停用	
有，曾用营养神经类药物（如甲钴胺、银杏叶制剂等），已停用	
有，其他类药物，已停用	
有，正在使用	

◎以下为耳鸣残疾量表（THI），共 25 个项目，该量表有助于从多维度更具体地了解您的耳鸣严重程度，请根据条文内容，如实进行选择。

第 23 题　耳鸣残疾评估量表（THI）　　[矩阵量表题]

题目\选项	有	有时候	没有
1 耳鸣会让你难以集中注意力吗？			
2 耳鸣声会影响你听他人的声音吗？			
3 耳鸣声会使你感到困惑吗？			
4 耳鸣声会影响你入睡吗？			
5 耳鸣声是否影响你享受社会活动？（比如外出就餐，看电影等等）			
6 耳鸣是否影响你享受生活？			
7 耳鸣是否干扰你的工作或家庭责任？			
8 耳鸣有没有影响你阅读？			
9 你是否很难不去想耳鸣而做其他事情？			
10 耳鸣是否让你很疲倦？			
11 当你有压力的时候耳鸣是否会加重？			
12 耳鸣是否让你有挫折感？			
13 你是否经常抱怨耳鸣？			
14 耳鸣声会使你生气吗？			
15 耳鸣有没有使你易发火？			
16 耳鸣是否让你没有安全感？			
17 耳鸣有没有让你很沮丧？			
18 你是否认为耳鸣让你和你的家人及朋友关系紧张？			
19 耳鸣是否让你感到压抑？			
20 耳鸣是否让你感到焦虑？			
21 你是否感到再也不能忍受耳鸣了？			
22 你是否认为无法控制耳鸣？			
23 耳鸣是否让你觉得患了很严重的疾病？			
24 你是否觉得自己无法摆脱耳鸣？			

25 耳鸣会让你感到绝望吗?			

依据 THI 总得分情况将耳鸣残疾分为 5 级:1-16 分为 1 级（轻微）；18-36 分为 2 级（轻度）；38-56 分为 3 级（中度）；58-76 分为 4 级（重度）；78-100 分为 5 级（灾难性）。

◎下面 10 个问题有助于了解您的近期（近一个月内）睡眠情况，请您在最符合自己的每个问题上选择一个答案。

第 24 题 您觉得平时睡眠足够吗？ [评分单选]

选项	
睡眠过多了	
睡眠正好	
睡眠欠一些	
睡眠不够	
睡眠时间远远不够	

第 25 题 您在睡眠后是否已觉得充分休息过了？ [评分单选]

选项	
觉得充分休息过了	
觉得休息过了	
觉得休息了一点	
不觉得休息过了	
觉得一点儿也没休息	

第 26 题 您平均每个晚上大约能睡几小时？ [评分单选]

选项	
≥9 小时	
7-8 小时	
5-6 小时	

3-4 小时	
1-2 小时	

第 27 题 睡眠情况矩阵题 [矩阵量表题]

题目\选项	0-5 天	很少（6-12 天）	有时（13-18 天）	经常（19-24 天）	总是（25-31 天）
您晚上已睡过觉，白天是否打瞌睡？					
您是否有入睡困难？					
您入睡后中间是否易醒？					
您在醒后是否难于再入睡？					
您是否多梦或常被恶梦惊醒？					
为了睡眠，您是否吃安眠药？					

第 28 题 您失眠后心情（心境）如何？ [评分单选]

选项	
无不适	
无所谓	
有时心烦、急躁	
心慌、气短	
乏力、没精神、做事效率低	

五个部分共 10 个项目，待自评结束后，把 10 个项目中的各项分数相加，即得到总分。总分范围为 10-50 分;总分数愈低,说明睡眠问题愈少；总分数愈高，说明睡眠问题愈重、愈多。此量表最低分为 10 分（基本无睡眠问题），最高分为 50 分（最严重）。

◎下面有二十条文字（括号中为症状名称），请仔细阅读每一条，把意思弄明白，每一条文字后有四级评分,分别表示：“1”--没有或很少时间；“2”--小部分时间；“3”--相当多的时间；“4”--绝大部分或全部时间。然后根据您**最近一星期**的实际情况，选择对应分数选项。

第 29 题　焦虑自评量表（SAS）　　[矩阵量表题]

题目\选项	1	2	3	4
1.我觉得比平时容易紧张和着急（焦虑）				
2.我无缘无故地感到害怕（害怕）				
3.我容易心里烦乱或觉得惊恐（惊恐）				
4.我觉得我可能将要发疯（发疯感）				
6.我手脚发抖打颤（手足颤抖）				
7.我因为头痛、颈痛和背痛而苦恼（躯体疼痛）				
8.我感觉容易衰弱和疲乏（乏力）				
10.我觉得心跳得快（心悸）				
11.我因为一阵阵头晕而苦恼（头昏）				
12.我有晕倒发作，或觉得要晕倒似的（晕厥感）				
14.我手脚麻木和刺痛（手足刺痛）				
15.我因胃痛和消化不良而苦恼（胃痛或消化不良）				
16.我常常要小便（尿意频数）				
18.我脸红发热（面部潮红）				
20.我做恶梦（恶梦）				

第 30 题　焦虑自评量表（SAS）　　[矩阵量表题]

题目\选项	1	2	3	4
5.我觉得一切都很好，也不会发生什么不幸（不幸预感）				
9.我觉得心平气和，并且容易安静坐着（静坐不能）				
13.我呼气吸气都感到很容易（呼吸困难）				
17.我的手常常是干燥温暖的（多汗）				
19.我容易入睡并且一夜睡得很好（睡眠障碍）				

SAS 所有条目得分之和为粗分，标准分为粗分乘以 1.25 后的整数部分。其中 SAS 评分小于 50 分为无焦虑，50-59 分为轻度焦虑，60-69 分为中度焦虑，70 分以上为重度焦虑。

SAS 的 20 个项目中，第 5, 9, 13, 17, 19 条，此 5 个项目的计分，必须反向计算，即“4”代表 1 分，“3”代表 2 分 ，“2”代表 3 分，“1”代表 4 分。

◎以下有 20 条文字，请根据您**近一周的感觉**来进行评分，数字的顺序依次为；“1”表示“从无”，“2”表示“有时”，“3”表示“经常”，“4”表示“持续”。

第 31 题 抑郁自评量表（SDS） [矩阵量表题]

题目\选项	1	2	3	4
1.我感到情绪沮丧，郁闷				
3.我要哭或者想哭				
4.我夜间睡眠不好				
7.我感到体重减轻				
8.我为便秘烦恼				
9.我的心跳比平时快				
10.我无故感到疲劳				
13.我坐卧不安，难以保持平静				
15.我比平时更容易激怒				
19.假若我死了别人会过得更好				

第 32 题 抑郁自评量表（SDS） [矩阵量表题]

题目\选项	1	2	3	4
2.我感到早晨心情最好				
5.我吃饭像平时一样多				
6.我的性功能正常				
11.我的头脑像往常一样清楚				
12.我做事情像平时一样不感到困难				
14.我对未来感到有希望				
16.我觉得决定什么事很容易				
17.我感到自己是有用的和不可缺少的人				

18.我的生活很有意义				
20.我仍旧喜爱平时喜爱的东西				

SDS 所有条目得分之和为粗分，标准分为粗分乘以 1.25 后的整数部分。SDS 评分小于 53 分为无抑郁，53-62 分为轻度抑郁，63-72 分中度抑郁, 73 分以上为重度抑郁。
SD 的 20 个项目中，第 2、5、6、11、12、14、16、17、18、20 条，此 10 个项目的计分，必须反向计算。

（非常感谢您的填表，祝您早日康复！）
