# Supplementary material for: Beyond the auditory: anxiety bridges sleep disturbances and depressive symptoms to tinnitus handicap
Source: Front Psychiatry. 2026 May 22;17:1830941. doi: 10.3389/fpsyt.2026.1830941 (PMC13236688; doi:10.3389/fpsyt.2026.1830941)
Supplement: Supplementary file 2 [file DataSheet2.pdf]

Survey and Self-Assessment Form for Patients with Tinnitus

Question 1: What is your treatment status? [Single-choice question]

option	
first visit	
One treatment course has been completed.	
Two treatment courses have been completed.	
Treatment continues beyond two completed courses	
Follow-up one month after treatment completion	
Follow-up at 3 months after treatment completion	

Question 2: What is your age? [Fill-in-the-blank question]

Question 3: How many specific treatment sessions did you undergo? [Fill-in-the-blank question]

Question 4: What is your gender? [Single-choice question]

option	
man	
woman	

Question 5: What is your marital status? [Single-choice question]

option	
unmarried	
married	
dissociaton	
bereft of one's spouse	

Question 6: How long has it been since the onset of your illness? [Single-choice question]

option	
less than 2 weeks	
2 weeks to 1 month	
1-3 months	
3-6 months	
7 months to 1 year	
1–5 years	
6-10 years	
Over 10 years (>10 years)	

Question 7: Do you experience unilateral tinnitus or bilateral tinnitus? [Single-choice question]

option	
No tinnitus	
Unilateral tinnitus	
Bilateral tinnitus	

Question 8: Do you currently experience symptoms of hearing loss? [Single-choice question]

option	
yes	
deny	

Question 9: Do you currently experience symptoms of vertigo? [Single-choice question]

option	
yes	
deny	

Question 10: Do you experience symptoms of headache? [Single-choice question]

option	
yes	
deny	

Question 11: Do you currently experience symptoms of ear fullness or pressure? [Single-choice question]

option	
yes	
deny	

Question 12: Do you have auditory hypersensitivity? [Single-choice question]

option	
yes	
deny	

Question 13: Have you ever had or currently have rhinitis? [Single-choice question]

option	
yes	
deny	

Question 14: Have you ever had or currently have otitis media? [Single-choice question]

option	
yes	
deny	

Question 15: Have you ever had or currently have hypertension? [Single-choice question]

option	
yes	
deny	

Question 16: Have you ever had or currently have diabetes mellitus? [Single-choice question]

option	
yes	
deny	

Question 17: Have you ever smoked or currently have a smoking habit? [Single-choice question]

option	
never	
Present, previously abstinent	
Still available now	

Question 18: Have you ever consumed alcohol or do you have a current drinking habit? [Single-choice question]

option	
never	
Present, previously abstinent	

Still available now	

Question 19: How long do you wear headphones? [Single-choice question]

option	
Never wear headphones	
The average daily headphone usage duration is ≤1 hour.	
The average daily headphone usage duration exceeds 1 hour.	

Question 20: What are the triggers for symptom onset? [Multiple-choice question]

option	
tired	
upper respiratory tract infection	
agitation	
Noise stimulation	
other	
No obvious cause	

Question 21: Are you exposed to a noisy environment for an extended period? [Single-choice question]

option	
yes	
deny	

Question 22: Have you used any medications for treatment since the onset of the disease? [Multiple Choice]

option	
not have	

Yes, previously used hormonal medications (e.g., dexamethasone), discontinued	
Yes, previously used neurotrophic drugs (e.g., mecobalamin, ginkgo biloba extract preparations), discontinued	
Yes, other classes of drugs, discontinued	
Yes, currently in use	

The following is the Tinnitus Hearing Impairment Scale (THI), consisting of 25 items. This scale facilitates a more multidimensional and specific assessment of your tinnitus severity. Please select the options truthfully based on the item descriptions.

Question 23: Tinnitus Disability Assessment Scale (THI) [Matrix Scale Item]

Title Options	have	between times	not have
1 Does tinnitus impair your ability to concentrate?			
2. Does tinnitus affect your ability to hear others' voices?			
3 Does tinnitus cause confusion?			
4. Does tinnitus affect your sleep?			
5 Does tinnitus affect your enjoyment of social activities (e.g., dining out, watching movies, etc.)?			
6 Does tinnitus affect your quality of life?			
7 Does tinnitus interfere with your work or family responsibilities?			
Does tinnitus affect your reading ability?			
9 Do you find it difficult to perform other tasks without thinking about tinnitus?			
10 Does tinnitus cause you significant fatigue?			
11 Does tinnitus worsen when you are under stress?			
12 Does tinnitus cause you a sense of frustration?			
13 Do you frequently complain of tinnitus?			
14. Does tinnitus sound make you angry?			
15 Does tinnitus make you prone to anger?			
16 Does tinnitus make you feel insecure?			

17 Has tinnitus caused you significant distress?			
18 Do you believe tinnitus causes tension in your relationships with family and friends?			
19 Does tinnitus cause you distress?			
Does tinnitus cause you anxiety?			
21 Do you feel that you can no longer tolerate tinnitus?			
22 Do you feel unable to control tinnitus?			
23 Does tinnitus make you feel that you have a serious illness?			
24 Do you feel unable to relieve tinnitus?			
Does tinnitus cause you to feel hopeless?			

Tinnitus disability is classified into 5 levels based on the total THI score: Level 1 (mild) for scores of 1-16; Level 2 (mild) for scores of 18-36; Level 3 (moderate) for scores of 38-56; Level 4 (severe) for scores of 58-76; and Level 5 (disastrous) for scores of 78-100.

The following 10 questions will help assess your recent sleep patterns (within the past month). Please select one answer for each question that best reflects your experience.

Question 24: Do you think you get enough sleep on a daily basis? [Scoring single-choice question]

option	
Excessive sleep	
Sleep is fine.	
Lack of sleep	
Insufficient sleep	
Sleep duration is significantly insufficient	

Question 25: Do you feel adequately rested after sleep? [Scoring: Multiple-choice question]

option	
I feel like I've had enough rest.	

I feel like I've had enough rest.	
I feel like I've had a bit of rest.	
I don't feel like I've had enough rest.	
I feel like I haven't rested at all.	

Question 26: How many hours do you sleep on average each night? [Scoring: Multiple-choice question]

option	
≥9 hours	
7-8 hours	
5-6 hours	
3-4 hours	
1-2 hours	

Question 27 Sleep Status Matrix Question [Matrix Scale Question]

Title Options	0-5 days	Rarely (6-12 days)	Sometimes (13-18 days)	Frequent (19-24 days)	Always (25-31 days)
You have slept at night, but do you nap during the day?					
Do you have difficulty falling asleep?					
Do you tend to wake up frequently during sleep?					
Do you have difficulty falling back asleep after waking up?					
Do you experience frequent dreaming or often wake up startled by nightmares?					
Do you take sleeping pills for sleep?					

Question 28: How does your mood (state of mind) change after insomnia? [Scoring: Multiple-choice question]

option	
No discomfort	
It doesn't matter.	
Occasional irritability and impatience	
Palpitation, shortness of breath	
Fatigue, lethargy, and reduced work efficiency	

The scale consists of five sections with a total of 10 items. After completing the self-assessment, the total score is calculated by summing up the scores of all 10 items. The total score ranges from 10 to 50 points; a lower total score indicates fewer sleep-related issues, while a higher total score suggests more severe and frequent sleep problems. The minimum score on this scale is 10 (indicating virtually no sleep issues), and the maximum score is 50 (indicating the most severe condition).

◎ Below are twenty items of text (symptom names are enclosed in parentheses). Please read each item carefully and comprehend its meaning. Each item is followed by a four-level scoring scale, indicating: "1" — no or very little time; "2" — a small portion of time; "3" — a considerable amount of time; "4" — the vast majority or all time. Based on your actual experience over the past week, select the corresponding score option.

Question 29 Anxiety Self-Rating Scale (SAS) [Matrix Scale Item]

Title Options	1	2	3	4
1. I experience increased nervousness and anxiety compared to usual.				
2. I feel fear (anxiety) without any apparent cause				
3. I am prone to mental distress or experience panic (panic disorder)				
4. I feel that I may be about to go mad (sense of impending madness)				
6. My hands and feet tremble (tremor of extremities)				
7. I am suffering from headaches, neck pain, and back pain (somatic pain)				
8. I feel prone to weakness and fatigue (lack of energy)				
10. I feel my heart beating rapidly (palpitations)				
11. I am troubled by episodes of dizziness (vertigo).				

12. I have a syncope episode or feel like I am about to faint (syncope sensation)				
14. Numbness and tingling in the extremities (paresthesia of hands and feet)				
15. I am suffering from stomach pain and dyspepsia (stomach pain or indigestion)				
16. I frequently experience urinary urgency (frequent urination).				
18. Facial flushing and warmth (facial erythema)				
20. I have nightmares.				

Question 30 Anxiety Self-Rating Scale (SAS) [Matrix Scale Item]

Title Options	1	2	3	4
5. I feel everything is fine and no unfortunate events will occur (unfortunate premonition)				
9. I feel calm and composed, and can easily sit quietly (meditation is not possible).				
13. I experience difficulty breathing during both exhalation and inhalation.				
17. My hands are often dry and warm (drenched in sweat).				
19. I fall asleep easily and sleep well throughout the night (sleep disorder)				

The total score of all SAS items represents the crude score, while the standardized score is the integer part obtained by multiplying the crude score by 1.25. Specifically, a SAS score below 50 indicates no anxiety, 50-59 indicates mild anxiety, 60-69 indicates moderate anxiety, and scores above 70 indicate severe anxiety.
Among the 20 items in SAS, items 5, 9, 13,17, and 19 require reverse scoring: '4' represents 1 point, '3' represents 2 points, '2' represents 3 points, and '1' represents 4 points.

◎ Below are 20 items. Please rate your symptoms based on your feelings over the past week. The numerical order is as follows: "1" indicates "never", "2" indicates "sometimes", "3" indicates "often", and "4" indicates "persistently".

Question 31: Self-Rating Depression Scale (SDS) [Matrix Scale Item]

Title Options	1	2	3	4
1. I feel emotionally depressed and melancholic				
3. I want to cry or feel like crying				

4. I have poor sleep at night				
7. I experienced weight loss				
8. I am troubled by constipation				
9. My heart rate is faster than usual.				
10. I feel fatigued without apparent cause				
13. I am restless and unable to maintain calmness.				
15. I am more easily angered than usual.				
19. If I were to die, others would have an easier life.				

Question 32: Self-Rating Depression Scale (SDS) [Matrix Scale Item]

Title Options	1	2	3	4
2. I feel my best mood in the morning				
5. I eat the same amount as usual.				
6. My sexual function is normal				
11. My mind is as clear as usual.				
12. I find doing things as usual not difficult.				
14. I feel hopeful about the future				
16. I find it easy to decide what to do.				
17. I feel myself to be a useful and indispensable person				
18. My life is very meaningful				
20. I still love what I used to love.				

The total score of all SDS items represents the crude score, while the standardized score is the integer part obtained by multiplying the crude score by 1.25. A SDS score below 53 indicates no depression, 53-62 indicates mild depression, 63-72 indicates moderate depression, and scores above 73 indicate severe depression.
For SD's 20 items, the scoring for items 2, 5, 6, 11,12,14,16,17,18, and 20 must be calculated in reverse order.

(Thank you for completing the form. Wishing you a speedy recovery!)
